# Annual Address

**Published:** 1906-02

**Authors:** Frank Holland

**Affiliations:** President.


### ANNUAL ADDRESS.

By DR. FRANK HOLLAND, President.

(Georgia Dental Society Paper.)

*Members of the Georgia State Dental Society, Ladies and Gentlemen:*—With feelings of congratulation and pleasure I greet you on this, the occasion of our Thirty-Seventh Annual Meeting. The honor you conferred upon me last year to preside over your deliberations at this session is most sincerely appreciated.

It shall be my honest endeavor to be fair and just to all, at the same time you must realize that unless the rules for our guidance, as laid down, are enforced, that the measure of success and dignity which we all desire cannot be attained.

I beg that you be patient and tolerant, and that your criticisms be tempered with mercy, allowing me credit for honesty of purpose and a strong desire for the welfare of our organization as a factor in upbuilding the profession of dentistry.

When we meet year after year I feel that whatever of good or ill has brought sunshine or cloud, we come to these meetings, not only for the purpose of improving ourselves professionally, but to greet old friends with that degree of social pleasure that makes life worth living, at the same time, felicitating ourselves upon meeting new ones. Our labors ended, we return with minds refreshed; having gained some sunshine, some new skill which beautifies the mind and makes us better appreciate life and its beauties, and increases our ability to minister to human suffering. Draper says: "The life of an individual is a miniature of the life of a nation," therefore, the foundation of success in every organization is due to individual effort. "Individual professional life is always indicative of what the professional whole will be." If the individual occupies a position which is not dependent upon persistent and systematic work for superiority, then it will undoubtedly be reflected in the life of any organization of which he is a member and to its disadvantage. In the beginning, then, our advancement must always be born of individual worth. There is not one among us who cannot testify that this is true. If he has had success in different fields, tracing back you will find it came through personal effort, loyalty and enthusiasm. In our profession the careful student, the faithful worker, the enthusiast has made the entire body indebted to individual worth and force. Our Society meetings are made instructive and interesting by a few, and to their work our advancement is due. How shall we stimulate a desire for greater activity in our meetings? It is evident that only through self-culture can this be accomplished. The idea among the younger members is that individual force is given only to a few, when in truth it is given to every one who works for it. "The Temple of Knowledge is in our very midst, anyone may enter it who chooses."

### OUR RELATIONSHIP TO THE MEDICAL FRATERNITY.

At frequent intervals, in the journals have appeared lengthy articles on the subject of dentistry as a profession and its relationship to medicine. Writers and speakers have been and are still contending for the complete recognition of dentists by other ethical branches. Reasons for this unqualified alliance are given at length, and while the arguments seem to be potent, at the same time, in most instances, the real difficulties preventing as complete a coalition as might be desired are rarely touched upon.

From the standpoint of the idealist, it would seem feasible and entirely proper, that the ethical dentist, and one of attainment in the profession, should have equal standing with the practitioner of general medicine; but the fact that the subject admits of discussion, and the further fact that few in the medical ranks are ready to extend greetings to the dental body, goes to show that there is a disease somewhere in the situation, and a condition preventing assimilation.

As stated in the outset, apparently valid reasons have often been given to substantiate the claim of the dentist to full recognition by the older school of medicine. These reasons have in the main been theoretical, and the essayist or speaker has entirely ignored the obstacles which stand eternally as a practical bar to union. By the term practical, I mean every-day, common, tangible and well-known reasons that are constantly in evidence, and from which the dental body must entirely separate itself, before it can take the high rank sought for, and as yet not attained. Here and there, in nearly every community, is to be found a dentist who measures up entirely to the high standard set by his medical brethren. He is in the first place, honest towards himself and patients (which, by the way, is the first step, the fundamental principle, and the basis of ethical deportment). Further, he is a student not only of the things which belong exclusively to his particular profession, but his mind by constant training, and as constant use, has broadened to such a degree, that he is enabled not only to play a positive part in general scientific affairs, but in addition, is a recognized active force in his community, and the world at large. To such a man we are to look for the answer to the query: How can the great body of professional dentists become thoroughly allied with the medical body without apparent effort or discussion, and what are the present obstacles in the way of such a union? The answer can be given quickly and in brief, by asking another question, which will bring us at once to the point at issue. Where in all your experience have you found a man occupying such a position as the one just described, who has ever been guilty of bringing his practice down to a purely commercial basis? Without dwelling on the subject longer, we know full well that such a man does not exist in the dental ranks. Show me a man who has made progress in his profession, whose word touching any question has weight the world over, whose presence adds dignity to any assembly of dentists, and I will show you one who has never in all his professional career allowed his practice to sink to such a level.

Upon the other hand, the dentist who fools himself by thinking he can by employment of inferior methods and cheap materials, and by so doing keep the patient in a state of blissful ignorance, and, at the same time, satisfied with the treatment, is simply preparing the way for a life of comparative obscurity, and one in which the element of poverty will always lurk.

Is this class on the increase? Unfortunately, yes! And in this positive statement lies the answer to the question first propounded, for it is true beyond a doubt, that in spite of all theories, the medical profession will never agree to meet on equal terms with men of a so-called allied profession, who have within their ranks such a preponderance of members, who are seemingly devoid of the most necessary qualities that make for professional and ethical standing.

MEMBERSHIP.

Our Society has done a good work in the past, but that we have been negligent in some things is quite apparent. Have we looked after our membership as we should have done, and is the number what it should be? Have we used the proper efforts to secure a larger membership? There are between five and six hundred dentists in Georgia. Out of this number there must at least be three hundred eligible to membership in our Society, as yet we have only 125. That numbers increase the importance and influence of any organization or profession, no one will deny, and for that reason I would suggest that we formulate some plan to increase our membership. The medical body of our State has a special committee on membership which has done good work in the past few years. Why should we not have such a committee?

RECOMMENDATIONS.

I would suggest that we appoint three prominent members of our Society, whose duty it shall be to ascertain from the Dental Directory those who are entitled to membership. In many of the large, as well as smaller places, they will find a member of our Society, and perhaps one or more ethical practitioners, who are not members, but who, if approached properly, would gladly join our ranks and become valuable members. If this special committee would by correspondence with the member induce him to approach his brother in the profession upon the importance of becoming a member, it would not only bring about a more cordial feeling between them, but have the effect of bringing the indifferent into our ranks. We should have at least 250 in the Georgia State Dental Society, and within a short time this number can be secured, if sincere effort and activity is resorted to.

### OUR INDIVIDUALITY.

All fraternal and civic organizations have adopted some badge, button or embossed certificate of membership, with some appropriate motto inscribed theron. I would, therefore, suggest the appointment of a committee of three to select a suitable membership card and motto, with proper spaces for the inscription of the name of the member. These cards should be made attractive. Properly framed and suspended in the office, they would at once show our relation to the profession as that of an ethical one. Let it be positively understood that such certificate shall be the pr .perty of the Society, and any member violating the code of ethics or in any manner forfeiting his right to membership in th Society, will forfeit the right of possession.

### SPECIAL ESSAYIST.

Another important thought I desire to call your attention to, is that of making our meetings more attractive. I would, therefore, recommend that a special committee of two be appointed whose duty it shall be to endeavor to procure some distinguished member of our profession to be the special essayist. Have him write a paper on some scientific subject, select two or three members to discuss the paper, after having an opportunity to look over it; in other words, to have an intelligent discussion of the subject would not only add largely to the interest of the meeting, but would be profitable to all. It should be the special purpose of the Society to have as full line of clinics as possible, and to have clinicians who are capable.

### FEES FOR PROFESSIONAL SERVICES—PROPER COMPENSATION.

I would not presume to suggest ways and means, but I am constrained at this opportune time to counsel our brethren with regard to their business relationship, as it pertains to their profession. There is no question but that dentists are more poorly re-imbursed for their services than any class of professional men, and while this is true, I am convinced that the fault lies wholly at our doors. The public at large never places a more exalted estimate on a man than he deserves, or than he places upon himself. The world's respect and opinion is gauged by one's own self-respect and conduct. As this may apply to one aspect of our lives, so will it to all. It is a deplorable fact that a large per cent. of the time of the otherwise busy dentist is consumed in making examinations, treating teeth, and preparing the mouths of patients in various ways, leading up to the final operation, without even making any appreciable charge for services. Many dentists allow themselves to be imposed upon by prominent and high-salaried men in the clergy, without ever making a charge. Why should we not charge a minister of the Gospel, who is receiving one to three thousand dollars per annum, the same as we would other salaried men who may not have an income equal to the above mentioned individual. A dentist should not feel himself obliged to practice for every physician and family in the community free, yet most of them do it.

Finally, the world usually responds to its financial obligations with about the same degree of promptness as is manifested by the individual. If you perform a professional service and wait sixty days before sending your bill, the chances are that you will then wait ninety days before receiving a check.

The specialist charges for every treatment, and his services are accordingly valued. Why should we, as dentists, allow a depreciated estimate to be placed upon a service of relatively the same importance rendered by us, by doing it gratis? Charge for all professional attendance, and send your bills promptly every thirty days. You will soon educate the public to place a proper value on your services, and that you expect adequate compensation in return. Be prompt to pay your preacher and physician, and exact from them equal business-like treatment. They will respond, and respect you accordingly.

